# Supplementary material for: Distinct Dopamine D2 Receptor Antagonists Differentially Impact D2 Receptor Oligomerization
Source: Int J Mol Sci. 2019 Apr 4;20(7):1686. doi: 10.3390/ijms20071686 (PMC6480712; doi:10.3390/ijms20071686)
Supplement: Supplementary file 1 [file ijms-20-01686-s001.pdf]

Supplementary Materials

Distinct Dopamine D_2 receptor antagonists differentially impact D_2 receptor oligomerization

Elise Wouters[1], Adrián R. Marín[2,3,4], James A. R. Dalton[2,3,4], Jesús Giraldo[2,3,4], Christophe Stove[1*]

[1] Laboratory of Toxicology, Department of Bioanalysis, Faculty of Pharmaceutical Sciences, Ghent University, Ottergemsesteenweg 460, 9000 Ghent, Belgium
[2] Laboratory of Molecular Neuropharmacology and Bioinformatics, Unitat de Bioestadística, Institut de Neurociències, Universitat Autònoma de Barcelona, 08193 Bellaterra, Spain
[3] Instituto de Salud Carlos III, Centro de Investigación Biomédica en Red de Salud Mental, CIBERSAM, Universitat Autònoma de Barcelona, 08193 Bellaterra, Spain
[4] Unitat de Neurociència Traslacional, Parc Taulí Hospital Universitari, Institut d'Investigació i Innovació Parc Taulí (I3PT), Institut de Neurociències, Universitat Autònoma de Barcelona, 08193 Bellaterra, Spain
* Correspondence: christophe.stove@UGent.be; Tel.: +32 9 264 81 35

List of contents

Figure S1. Signals obtained following incubation of $D_{2L}R$-LgBiT and $D_{2L}R$-SmBiT transfected HEK293T cells with D_2R antagonists for 10 min ………………...3

Figure S2. Signals obtained following incubation of $D_{2L}R$-LgBiT and $D_{2L}R$-SmBiT transfected HEK293T cells with D_2R antagonists for 30 min ………………..……………………..…......3

Figure S3. Signals obtained following incubation of NanoLuciferase transfected HEK293T cells with D_2R antagonists for 16 h…………………………………………….………….........3

Figure S4. MD simulations conformational stability and D_2R homodimer interaction changes……………………………………………………………………………………....4

Figure S5. Crystallized orthosteric binding pose of risperidone……………………………....5

Figure S6. MD simulation selected Tyr199$^{5.48}$ and Phe390$^{6.52}$ $\chi 1$ dihedral angle…………..…...6

Figure S1

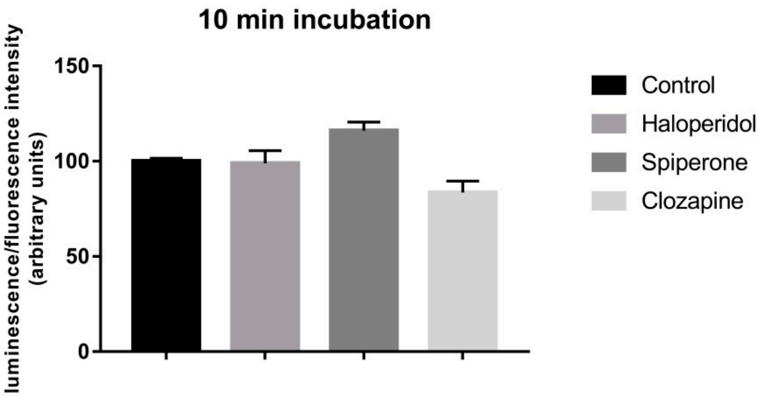

Figure S1. Signals obtained following incubation of $D_{2L}R$-LgBiT and $D_{2L}R$-SmBiT transfected HEK293T cells with D_2R antagonists haloperidol, spiperone and clozapine (10 μM) for 10 min. Control = solvent-treatment (DMSO≤0.1%). No significant effect was observed (n=3, ±SEM).

Figure S2

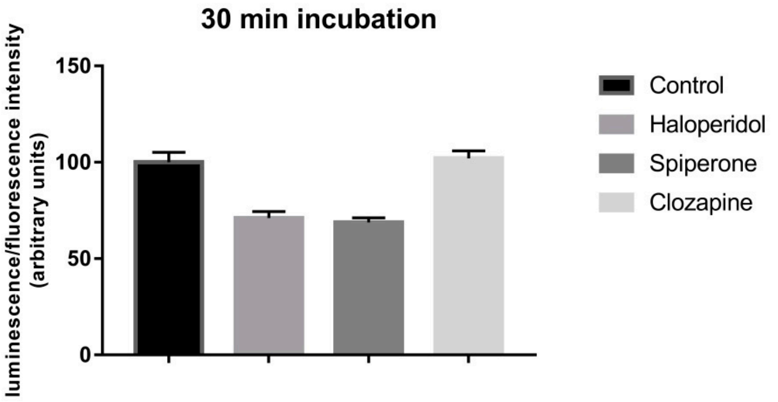

Figure S2. Signals obtained following incubation of $D_{2L}R$-LgBiT and $D_{2L}R$-SmBiT transfected HEK293T cells with D_2R antagonists haloperidol, spiperone and clozapine (10 μM) for 30 min. Control = solvent-treatment (DMSO≤0.1%). No significant effect was observed (n=3, ±SEM).

Figure S3

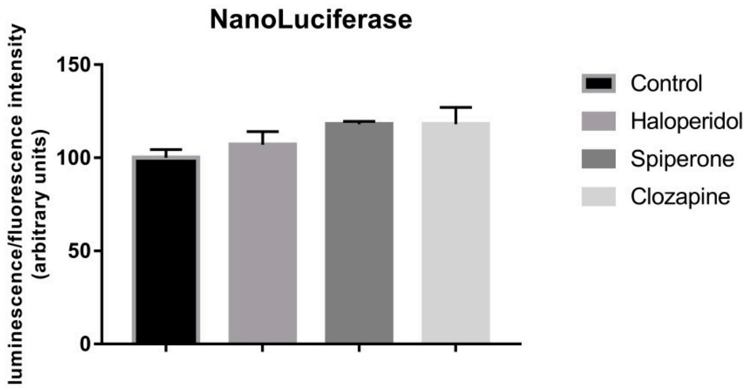

Figure S3. Signals obtained following incubation of NanoLuciferase transfected HEK293T cells with D_2R antagonists haloperidol, spiperone and clozapine (10 μM) for 16 h. Control = solvent-treatment (DMSO≤0.1%). No significant effect was observed (n=2, ±SEM).

Figure S4

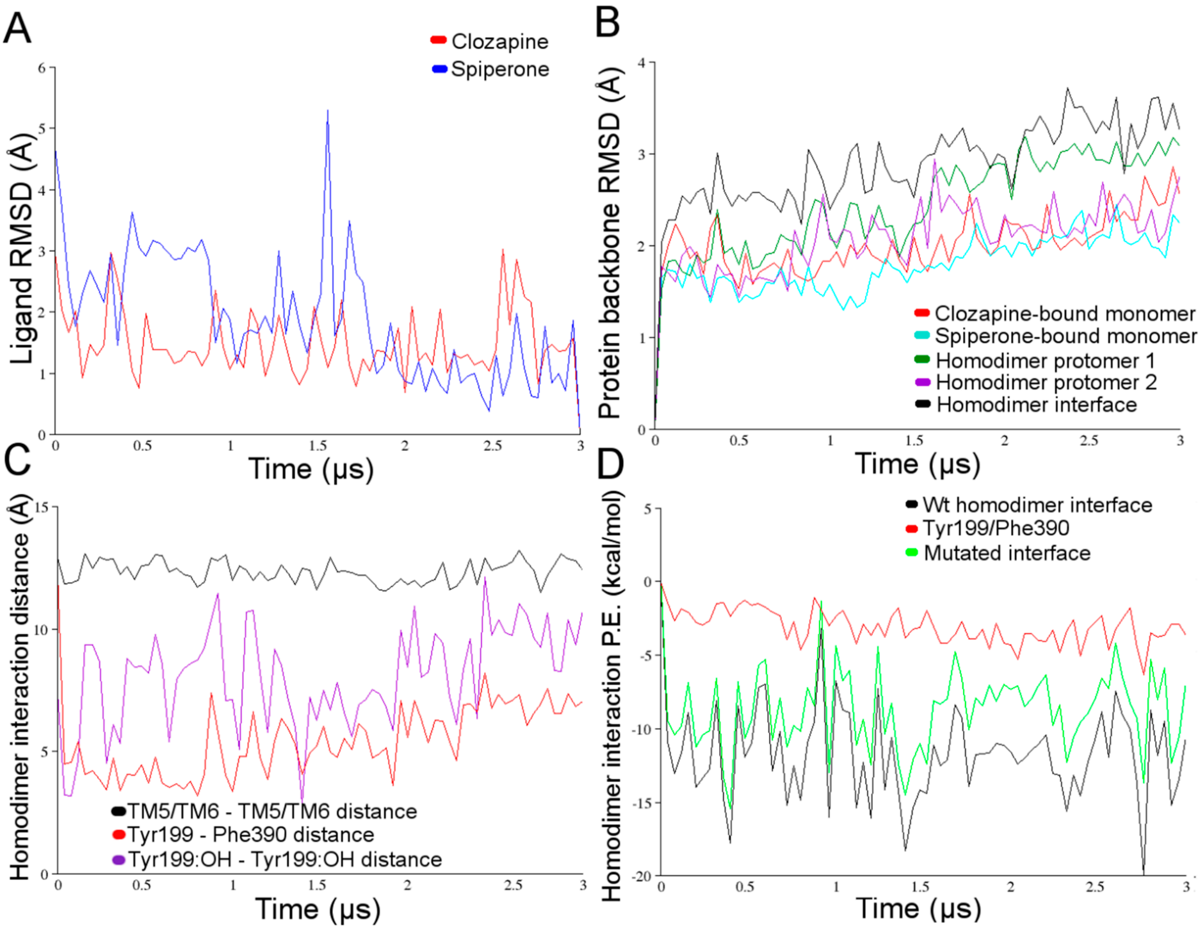

Figure S4. (A) Conformational stability of bound clozapine or spiperone (red and blue, respectively) in terms of RMSD compared against last conformation achieved during MD simulations. (B) Conformational change of the backbone of transmembrane domain of D_2R monomer with bound clozapine or spiperone, and protomer 1 or 2 and TM5-TM6-TM5-TM6 interface of D_2R homodimer (red, blue, purple, green and black, respectively) compared against initial conformation. (C) Distance between center of mass (COM) of interacting transmembrane helices (TM5 and TM6, in black), closer distance between residues Tyr199$^{5.48}$ and Phe390$^{6.52}$ (in red), and distance between sidechain oxygen atoms of Tyr199$^{5.48}$ of both protomers (in purple). (D) Energetic analysis of *wt* TM5-TM6-TM5-TM6 D_2R homodimer interface, specific energetic contribution of interactions between Tyr199$^{5.48}$ and Phe390$^{6.52}$, and mutated D_2R homodimer interface (Tyr199$^{5.48}$ and Phe390$^{6.52}$ replaced with alanine), coloured in black, red and green, respectively.

Figure S5

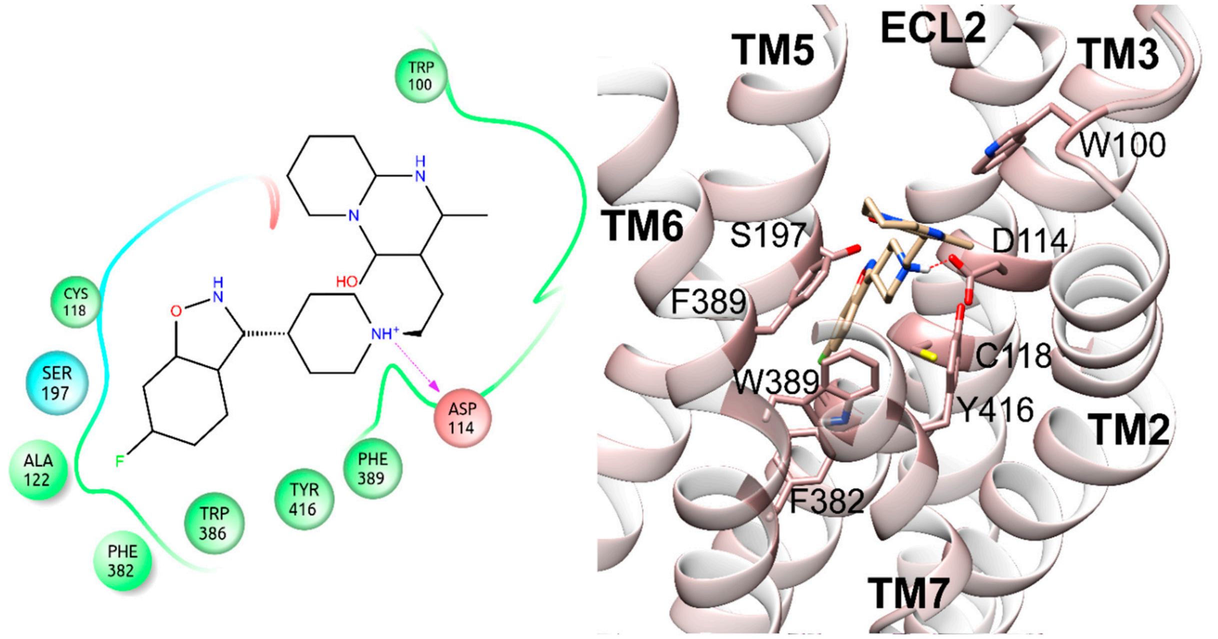

Figure S5. Crystallized orthosteric binding pose of risperidone. 2D and 3D binding pose defined by residues close-contacted (<3.5 Å) by risperidone (tan) in D2R crystal structure (brown, PDBid: 6CM4).

Figure S6

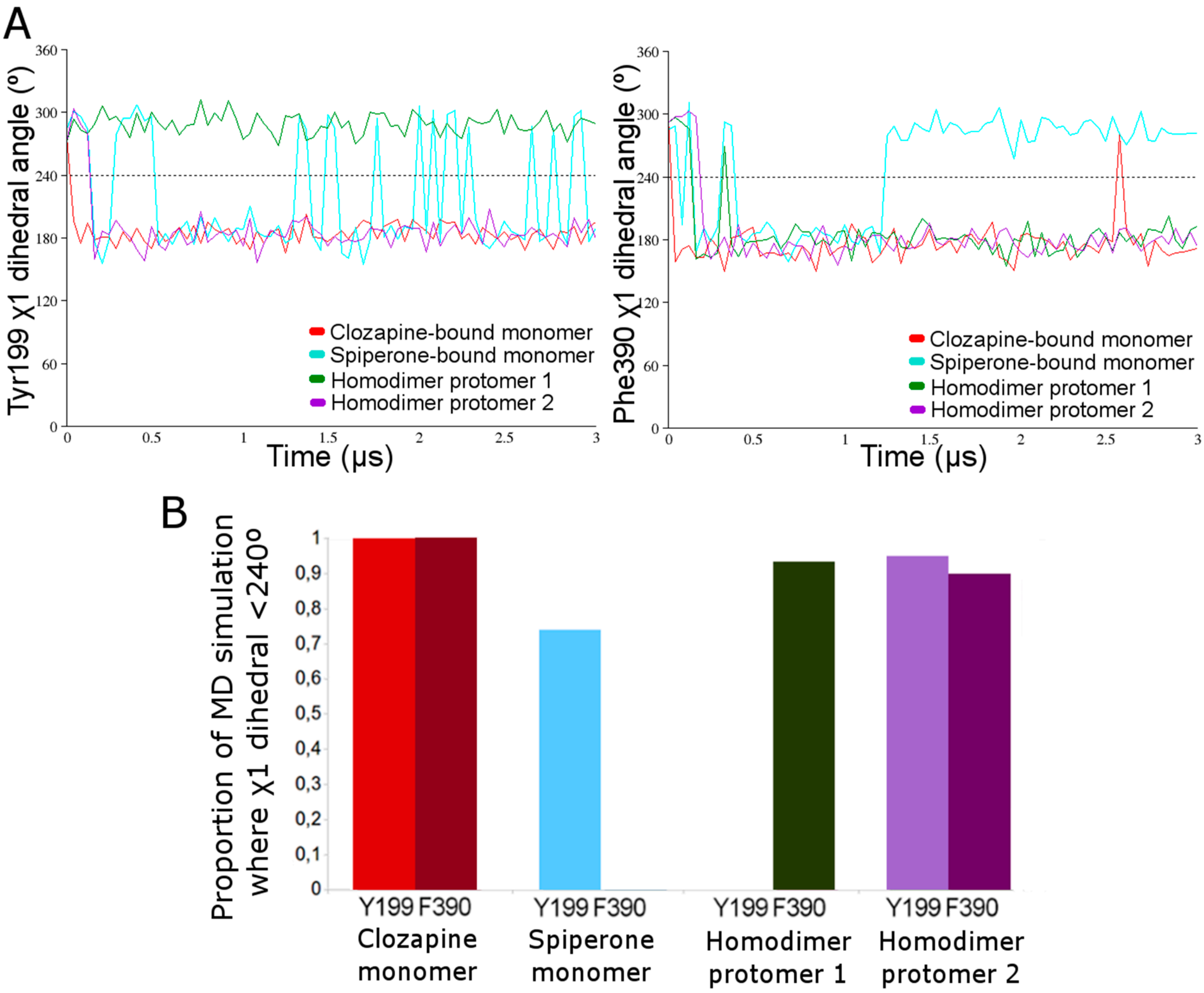

Figure S6. (A) Time-dependent plots of Tyr199$^{5.48}$ and Phe390$^{6.52}$ χ1 dihedral angle induction (left and right graph, respectively) of D_2R monomer with bound clozapine or spiperone, and protomer 1 or 2 of D_2R homodimer (red, blue, green and purple, respectively) during respective MD simulations. Black dotted line indicates *cis/trans* conformation threshold. (B) Proportion of selected χ1 dihedral angle conformation <240º in D_2R monomer with bound stable clozapine or spiperone, and protomer 1 and 2 of D_2R homodimer (red, blue, green and purple, respectively).
